# Distribution of incubation periods of COVID-19 in the Canadian context

**DOI:** 10.1038/s41598-021-91834-8

**Published:** 2021-06-15

**Authors:** Subhendu Paul, Emmanuel Lorin

**Affiliations:** 1 School of Mathematics and Statistics, Carleton University, Ottawa, K1S 5B6 Canada; 2 Centre de Recherches Mathématiques, Université de Montréal, Montréal, H3T 1J4 Canada

**Keywords:** Diseases, Health care, Health occupations, Medical research, Mathematics and computing

## Abstract

We propose a novel model based on a set of coupled delay differential equations with fourteen delays in order to accurately estimate the incubation period of COVID-19, employing publicly available data of confirmed corona cases. In this goal, we separate the total cases into fourteen groups for the corresponding fourteen incubation periods. The estimated mean incubation period we obtain is 6.74 days (95% Confidence Interval(CI): 6.35 to 7.13), and the 90th percentile is 11.64 days (95% CI: 11.22 to 12.17), corresponding to a good agreement with statistical supported studies. This model provides an almost zero-cost computational complexity to estimate the incubation period.

## Introduction

The outbreak of coronavirus disease 2019 (COVID-19), reported early in Wuhan (China) and spread around the world^1^, is creating dramatic and daily changes with profound impacts worldwide. People with underlying medical condition, respiratory disease, diabetes, cancer, etc., and older people are vulnerable to severe complications and death from coronavirus, although we are discovering new features of COVID-19 every day. In the absence of vaccination and proper medication, we can merely obey some non-pharmaceutical containments, lockdown, social distancing, hand hygiene, face masking, mobile app to trace corona-positive individuals, to help prevent the spread of this infectious disease. To minimize transmission of the virus through human-to-human interaction, quarantine of individuals with exposure to infectious pathogen is an effective strategy for containing contagious diseases. To govern a quarantine period for asymptomatic as well as presymptomatic individuals, it is essential to fully understand the incubation period of COVID-19 disease.

*The incubation period* of an infectious disease is the *time between when a person is infected by a virus and when the first symptoms of the disease are noticed.* Estimates of the incubation period for COVID-19 range from 2 to 14 days, according to the investigation so far. Precise knowledge of the incubation period is crucial to control infectious disease like COVID-19; a long incubation period means a high risk of further spreading the disease. The distribution of the incubation period can be used to estimate the basic reproduction number $R_0$, a key factor of epidemics, in order to measure the potential for disease transmission. It is indeed difficult to obtain a good estimate of the incubation period on the basis of limited data.

There are several statistical studies^2–12^, using a single measure^11^, estimated the incubation period of the current pandemic. In addition to those statistical approaches, there are numerous analytical and computational studies based on mathematical models, involving Ordinary Differential Equations (ODE)^13–21^ as well as Delay Differential Equations (DDE)^22–27^, to calculate the basic reproduction number $R_0$ and understand the underlying dynamics of the epidemic. Researchers usually consider a single delay models, occasionally two delays.

To the best of our knowledge, we are proposing for the first time a mathematical model, comprising fourteen delays, to estimate the incubation period utilizing publicly available data of the total number of corona-positive cases. This approach is free from any special type of samples in order to produce the distribution of the incubation period. It is then almost cost free, as it only involves a small scale computations. After a single calculation employing this method, we can generate the current distribution as well as previous distributions of the incubation period. We can also observe the change in the incubation period. In the statistical based approach, it is usually difficult to consider a large incubation period if the sample size is small. However, in this approach, we can go well beyond 14 days, the maximum incubation period we have set for the current work. In this context, we demonstrate the incubation period of the COVID-19 epidemic in Canada employing publicly available data of confirmed corona-positive cases^28^ . As of November 7, 2020, the World Health Organization (WHO) had confirmed a total of 251,338 cases of COVID-19 in Canada, including 10,381 deaths^1^.

There are several studies on incubation period mainly based on Chinese patients that can only provide a rough estimation for rest of the world. The incubation period may depends on age^29^ (median-age/country), hard immunity, public health system, corona testing capacities, daily corona cases, etc. For a better estimation of the incubation period for a particular region, we need to study local patients. Data collection is a bottleneck in studying the incubation period. However, one can easily estimate the incubation period using the approach we propose and publicly available data of confirmed cases.

**Figure 1:**
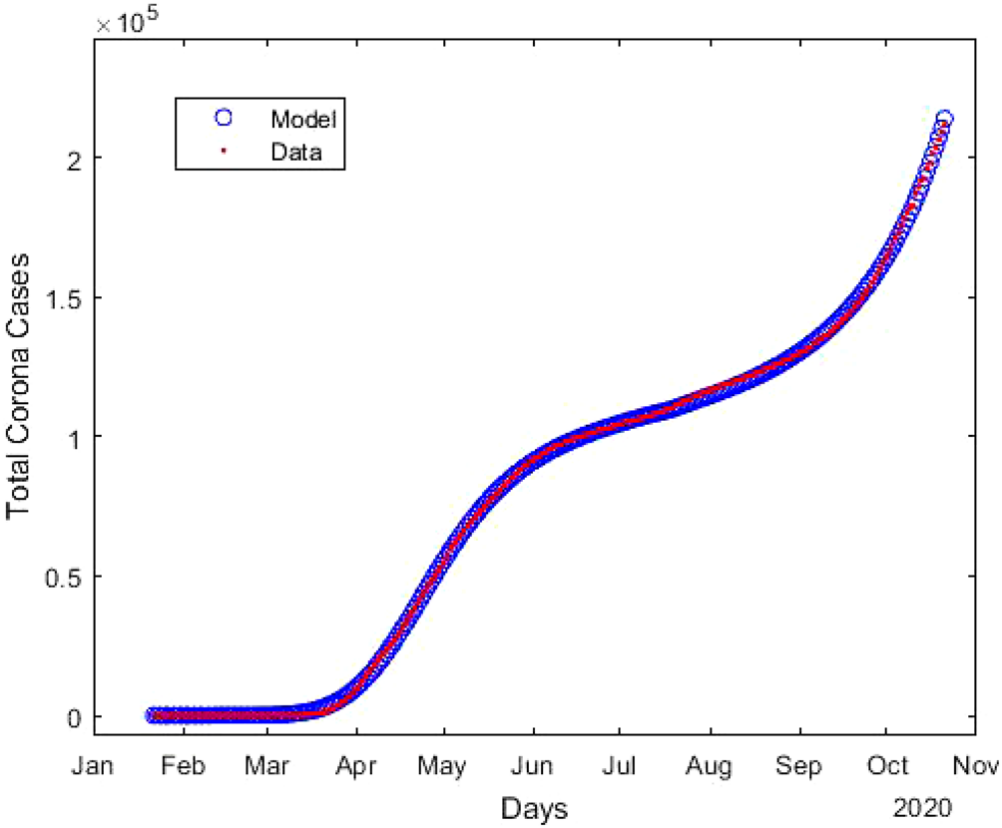
Estimation of the total number of confirmed coronavirus cases (*T*) compared to the available data^28^. Here blue circles indicates the results obtained from model, and red line is the publicly available data^28^.

**Figure 2:**
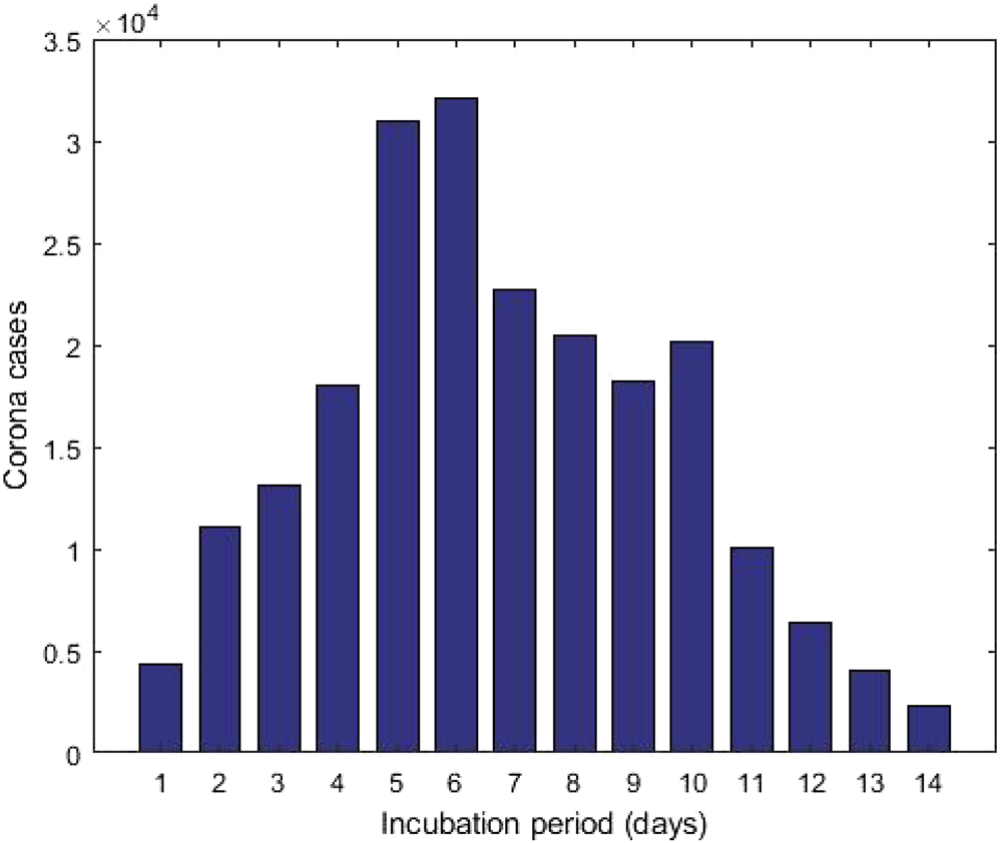
The cumulative data of confirmed corona cases as of October 23, 2020 is splitted into several incubation periods.

**Figure 3:**
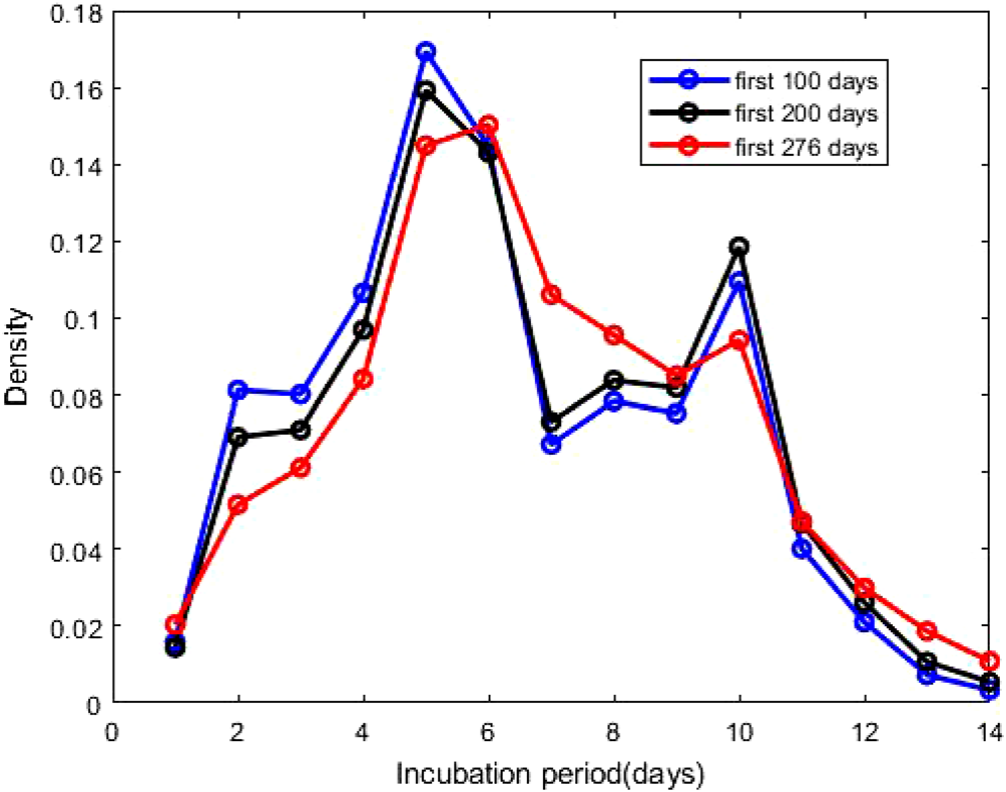
Probability densities of incubation period, presented in Eq. (1). The ’first 100 days’ indicates that the density of incubation period based on the cumulative data of the first 100 days during the epidemic starting from January 22, 2020 and similar for other two.

**Figure 4:**
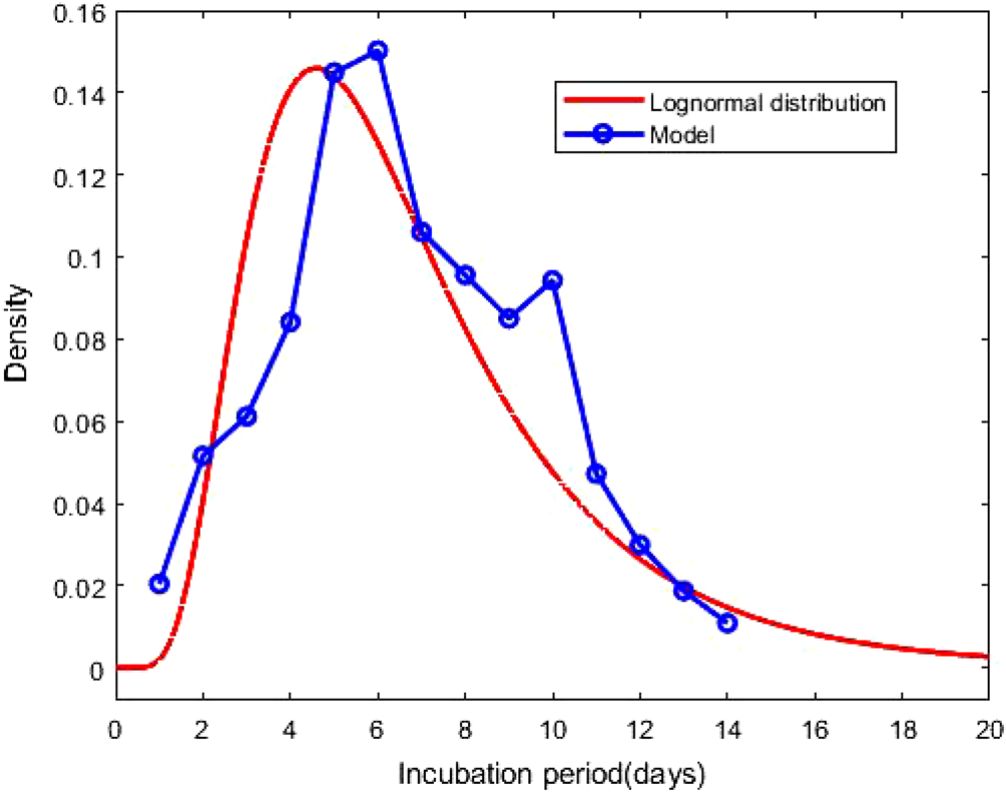
Probability density function of the lognormal distribution of the incubation period with $\mu = 1.79$ and $\sigma = 0.52$. The result based on the total confirmed corona cases of 276 days. The blue circles indicate the densities obtained from the model calculation.

**Figure 5:**
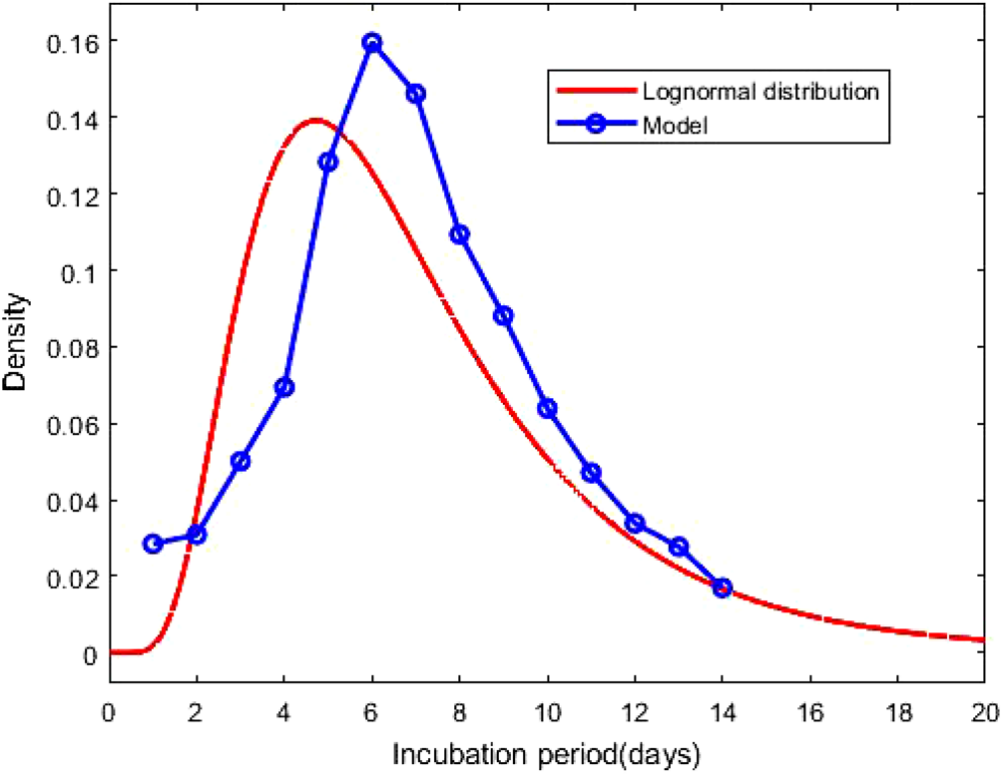
Probability density function of the lognormal distribution of the incubation period with $\mu = 1.83$ and $\sigma = 0.53$. The result based on the confirmed corona cases of a particular day, October 23, 2020. The blue circles indicate the densities obtained from the model calculation.

**Table 1:** A list of several studies along with sample size, mean and lognormal parameters $\mu$, $\sigma$.

Author	Data size	Mean (days)	$\mu$	$\sigma$
Backer et al.^2^	88	6.4	1.796	0.349
Lauer et al.^3^	181	5.5	1.621	0.418
Li et al.^4^	10	5.2	1.425	0.669
Bi et al.^5^	183	4.8	1.570	0.650
Jiang et al.^6^	40	4.9	1.530	0.464
Linton et al.^7^	158	5.6	1.611	0.472
Zhang et al.^8^	49	5.2	1.540	0.470
Ma et al.^9^	587	7.4	1.857	0.547
Leung et al.^10^	61	7.2	1.780	0.680
McAloon et al.^11^	Meta	5.8	1.63	0.50
Jing et al.^12^	1084	8.29		
Math model (raw data)	211,735	6.89		
Math model (lognormal)	211,735	6.7	1.788	0.520
Math model (raw data)	2587	7.17		
Math model (lognormal)	2587	7.0	1.827	0.528

## Results and discussion

After estimating the model parameters with sufficiently small values of the error functions, we compare in Fig. 1 total corona cases calculated with our model and the available data^28^. This shows excellent agreement. In Fig. 2, the confirmed cases 211,735 of 276 days are divided into fourteen groups. The *i*th compartment $T_i$, defined in Eq. (3), is the confirmed cases of 276 days corresponding to the incubation period of *i* day(s) for $i = 1, 2, \ldots , 14$. In addition $T_i$ is the frequency of the incubation period of *i* day(s), and using the bar chat, we obtain a mean incubation period of 6.89 days, a median of the incubation period of 6 days, 90th percentile of 11 days, 95th percentile of 12 days and 99th percentile of 13.5 days. The bar chat shows that mode of the incubation period is of 6 days, and there is a second peak for the incubation period at 10 days. However, the second peak is strongly dominated by the first one. From the bar chat presented in Fig. 2, we can also obtain the probability densities of incubation periods of the first *k* days during the epidemic, the total confirmed cases of the first *k* days starting on January 22, 2020. The probability densities $p_i^{(k)}$ of the first *k* days and corresponding incubation period *i* days for $i = 1, 2, \ldots , 14$ can be defined as

1 $$\begin{aligned} p_i^{(k)} = \frac{T_i^{(k)}}{\sum _{i = 1}^{14}T_i^{(k)}} \, , \end{aligned}$$

where $T_i^{(k)}$s are defined in Eq. (3). The probability densities $p_i^{(100)}$, $p_i^{(200)}$ and $p_i^{(276)}$ for $i = 1, 2, \ldots , 14$ are presented in Fig. 3. The density curves for the first 100, 200 and 276 days are similar, and the densities obey a remarkable configuration: for 2 $\le i \le$ 5, $p_i^{(100)}> p_i^{(200)} > p_i^{(276)}$, for 7 $\le i \le$ 9 and 11 $\le i \le$ 14 , $p_i^{(100)}< p_i^{(200)} < p_i^{(276)}$; although the second peak, for $i = 10$, does not follow the decreasing-increasing convention. For the incubation period of 10 days $p_{10}^{(200)}> p_{10}^{(100)} > p_{10}^{(276)}$, an oscillatory behaviour is observed. The descending pattern for 2 $\le i \le$ 5 and the ascending order for 7 $\le i \le$ 9 and 11 $\le i \le$ 14 indicate that the mean incubation period is rising. Now, we fit the frequency data for the first 276 days, presented in the bar chat Fig. 2, with the lognormal distribution function and obtain the lognormal distribution parameters $\mu = 1.79$ and $\sigma = 0.52$. Fig. 4 shows the lognormal distribution function of the incubation period for the first 276 days and population size 211,735 with $p_i^{(276)}$ for $i = 1, 2, \ldots , 14$. The estimated incubation period, obtained using lognormal distribution, has a mean of 6.74 (95% CI: 6.35 to 7.13), and the 90th percentile is 11.64 days (95% CI : 11.22 to 12.17). In addition, we focus on the distribution of the incubation period for a single day, October 23, 2020 which is the 276th day of the epidemic, with 2258 confirmed cases. The probability density ${\hat{p}}_i^{(k)}$ for a single day can be calculated as

2 $$\begin{aligned} {\hat{p}}_i^{(k)} = \frac{T_i^{(k)} - T_i^{(k-1)}}{\sum _{i = 1}^{14}T_i^{(k)} - \sum _{i = 1}^{14}T_i^{(k-1)}} \, , \end{aligned}$$

where $T_i^{(k)}$ is defined in Eq. (3). The estimated incubation period, obtained from the frequency table of 276th day and population size of 2258, has a mean of 7.14 days, a median of 7 days, the 90th percentile of 11 days, 95th percentile of 12.5 days and 99th percentile of 14 days. We generate the lognormal distribution function from the 276th day’s frequency data and obtain the lognormal distribution parameters $\mu = 1.83$ and $\sigma = 0.53$. Fig. 5 shows the lognormal distribution of 276th day along with ${\hat{p}}_i^{(276)}$. The estimated incubation period of 276th day, population size of 2258 and obtained using a lognormal distribution, has a mean of 6.98 days (95% CI: 6.41 to 7.55) and the 90th percentile of 12.29 days. A list of several studies along with the present calculation “Math.-Model” are presented in Table 1; we present the mean incubation period for the raw data as well as the lognormal distribution function. The list shows that our calculated data are closed the value reported by Ma *et. al.*^9^ using a larger sample size of 587. The calculated mean incubation period using two different ways, the raw data as well as the lognormal distribution are indeed closed, indicating that the raw data calculated using our mathematical model, are statistically significant for a lognormal distribution (statistical *p* value less than 0.001). It follows from the “Math.-Model” calculation, presented in Table 1, that the mean incubation period of 276th day, population size 2258, is greater than the mean incubation period of 276 days, population size 221,735 which demonstrates that the mean incubation period of COVID-19 is slightly increasing with time.

## Methods

In this section, we introduce a compartment based infectious disease model including a total of seventeen partitions, Lockdown, Susceptible, Infected and fourteen compartments of Total confirmed cases (LSIT). The model is constructed as a set of coupled delay differential equations involving several variables and parameters.

### The model

Modeling the spread of epidemic is an essential tool for projecting its outcome. By estimating important epidemiological parameters using the available database, we can make forecasts of different intervention scenarios. In the context of compartment based model, where the population of a region is distributed into several population groups, such as susceptible, infected, total cases, etc., is a simple but useful tool to demonstrate the panorama of an epidemic.

**Figure 6:**
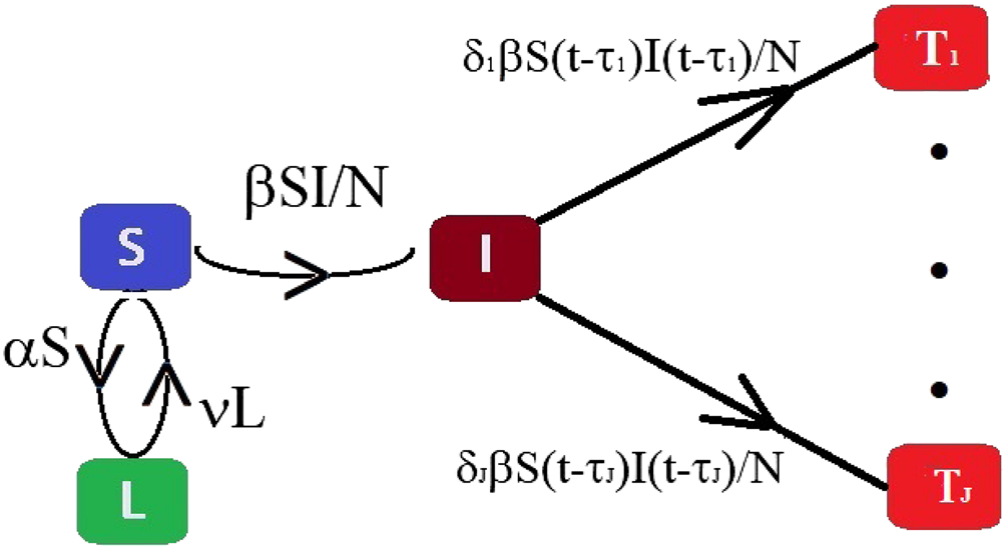
Schematic diagram of the compartmental based epidemic model, presented in Eq. (4).

In this article, we introduce an infectious disease model, extending the standard SIR model, including the phenomenon lockdown, a non-pharmaceutical way to prevent the spread of the epidemic. The schematic diagram of the model is presented in Fig. 6 with several compartments and various model parameters. The following are the underlying principles of the present model.

- The total population is constant (neglecting the migrations, births and unrelated deaths) and initially every individual is assumed susceptible to contract the disease.
- The disease is spread through the direct (face-to-face meeting) or indirect (through air current, common used or delivery items like door handles, grocery products) contact of susceptible individuals with the infected individuals.
- The quarantined area or the compartment for corona cases contains only members of the infected population who are tested corona-positive.
- The virus always kills some percent of the people it infects; the survivors percent represents the recovered group.
- There is a non-pharmaceutical policy (stay at home), commonly known as *lockdown*, to stop the spread of the disease.

Based on the above principles, we consider several compartments:

- Susceptible (*S*): the group of individuals who can be infected.
- Infected (*I*): the group of people who are spreading the contiguous disease.
- Total cases (*T*): the group of individuals who tested corona-positive (Active cases + Recovered + Deaths).
- Lockdown (insusceptible) (*L*): the group of persons who are keeping themselves safe.

In the model, we assume that there is no overlap between these two compartments, infected(*I*) and total cases (*T*). In other words, tested corona-positive individuals are assumed to be unable to substantially spread the disease due to isolation and are immune to re-infection after recovery^30^. The goal of the present model is to estimate the distribution of the incubation periods of COVID-19. In this goal, we split the compartment *T* into *J* subcomponents $T_1, \ldots , T_J$, where

3 $$\begin{aligned} T(t) = \sum _{i=1}^J T_i(t) \,\,\, \text {or}\,\,\, T^{(k)} = \sum _{i=1}^J T_i^{(k)}. \end{aligned}$$

In (3), *k* represents the time index and $T_i^{(k)}$ represents the total corona-positive cases corresponding the incubation period $\tau _i$, presented in Fig. 6. The time-dependent model is the following set of coupled delay differential equations:

4 $$\begin{aligned} \left\{ \begin{array}{lcl} \frac{dS}{dt} &{}=&{} -\beta (t) \frac{SI}{N} - \alpha (t) S + \nu (t) L \, , \\ \frac{dI}{dt} &{}=&{} \beta (t) \frac{SI}{N} - \sum _{i=1}^J\delta _i(t) \beta (t) \frac{S(t -\tau _i) I(t -\tau _i)}{N} \, , \\ \frac{dT_i}{dt} &{}=&{} \delta _i(t) \beta (t) \frac{ S(t -\tau _i) I(t -\tau _i)}{N} \, , \\ \frac{dL}{dt} &{}=&{} \alpha (t) S - \nu (t) L \, , \end{array} \right. \end{aligned}$$

where $\alpha (t)$, $\beta (t)$, $\delta _i(t)$, for $i = 1, \ldots , J$ and $\nu (t)$ are real positive parameters respectively modeling the rate of lockdown, the rate of infection, the rate of tested corona-positive corresponding the incubation period $\tau _i$ and the rate of transit from lockdown compartment to susceptible compartment, respectively. The coefficient $\alpha (t)$ can be expressed in the mathematical form as

$$\begin{aligned} \alpha (t) = \Big \{\begin{array}{c} \alpha ^{(1)}, \,\, t_0 \le t \le t_d \\ \alpha ^{(2)}, \,\, t_d < t \le t_1 \end{array} \end{aligned}$$

where $\alpha ^{(1)}$ and $\alpha ^{(2)}$ are constants, and the similar expressions are valid for other time-dependent parameters. Here $t_0$, $t_d$ and $t_1$ are the initial, intermediate and final times, respectively. The variables $S(t - \tau _i)$ and $I(t - \tau _i)$ denote the cumulative data of $(t -\tau _i)$ days, i.e., total number of suspected and infected individuals of $(t - \tau _i)$ days. The factor $\delta _i(t)\beta (t)S(t - \tau _i)I(t - \tau _i)/N$ conveys the rate of individuals who were infected $\tau _i$ days ago. It follows from (4), that for any *t*

5 $$\begin{aligned} L(t) + S(t) + I(t) + T(t) = N \, , \end{aligned}$$

where *N* (constant) is the total population size.

We solve (4) using matlab inner-embedded function **dde23** with particular sets of model parameters. To solve the initial value problem (4), in the interval $[t_0, t_1]$, we consider $L(t_0)$, $S(t_0)$, $I(t_0)$ and $T(t_0)$ as follows:

6 $$\begin{aligned} \left\{ \begin{array}{lcl} L(t_0) &{}=&{} 0 \, ,\\ S(t_0) &{}=&{} N - L(t_0) - I(t_0) - T(t_0)\, , \\ I(t_0) &{}=&{} q \, , \\ T(t_0) &{}=&{} {\widetilde{T}}(t_0) \, , \end{array} \right. \end{aligned}$$

where ${\widetilde{T}}(t_0)$ is the available data at time $t_0$, and *q* is the initial value adjusting parameters. Initially, there is no lockdown individual so that we can consider $L(t_0) = 0$. It follows from (3) and (6) that

7 $$\begin{aligned} \sum _{i = 1}^{J} T_i(t_0) = T(t_0) = {\tilde{T}}(t_0) . \end{aligned}$$

In the present context ${\tilde{T}}(t_0) = 0$, since there were no corona-positive cases reported on January 22, 2020. As a consequence, we also take $T_i(t_0) = 0$ for $i = 1, 2, \ldots , J$.

### Parameter estimation of the model

We focus on the exponential growth phase of the COVID-19 epidemic in Canada; one can use the approach to estimate the incubation period distribution for any region affected by the infectious disease. The time resolved (daily updated) database^28^ provides the number of total corona-positive cases. The optimal values of ${\mathbf {p}}(t) = (q, \alpha (t), \beta (t), \delta _1(t), \ldots , \delta _J(t), \nu )^T$, that is the set of initial values and model parameters, is obtained by minimizing the root mean square error function $E({\mathbf {p}}(t))$, defined as

8 $$\begin{aligned} E({\mathbf {p}}(t)) = \frac{1}{M}\sqrt{ \sum _{k = 1}^{M} (T^{(k)}({\mathbf {p}}(t)) - {\widetilde{T}}^{(k)})^2 }\,\,\, , \end{aligned}$$

where ${\widetilde{T}}^{(k)}$ is the available data of total corona-positive cases on the particular *k*th day, and $T^{(k)}$ is the calculated results obtained from System (4). The integer *M*, used in (8), is the size of the data set. Due to the complexity of the error function, the minimization using the matlab function **fminsearch** requires a very large number of iterations.

**Table 2:** The estimated values of the model parameters for two different time domains defined in Eq. (9).

Parameters	Estimated value	Parameters	Estimated value
$\alpha ^{(1)}$	0.01376	$\alpha ^{(2)}$	0.00102
$\beta ^{(1)}$	0.44952	$\beta ^{(2)}$	0.73348
$\delta _1^{(1)}$	0.01331	$\delta _1^{(2)}$	0.02180
$\delta _2^{(1)}$	0.07014	$\delta _2^{(2)}$	0.02454
$\delta _3^{(1)}$	0.07104	$\delta _3^{(2)}$	0.04129
$\delta _4^{(1)}$	0.09705	$\delta _4^{(2)}$	0.05931
$\delta _5^{(1)}$	0.15866	$\delta _5^{(2)}$	0.11374
$\delta _6^{(1)}$	0.13964	$\delta _6^{(2)}$	0.14658
$\delta _7^{(1)}$	0.06694	$\delta _7^{(2)}$	0.13907
$\delta _8^{(1)}$	0.08066	$\delta _8^{(2)}$	0.10781
$\delta _9^{(1)}$	0.08007	$\delta _9^{(2)}$	0.08989
$\delta _{10}^{(1)}$	0.12072	$\delta _{10}^{(2)}$	0.06755
$\delta _{11}^{(1)}$	0.04584	$\delta _{11}^{(2)}$	0.05151
$\delta _{12}^{(1)}$	0.02497	$\delta _{12}^{(2)}$	0.03834
$\delta _{13}^{(1)}$	0.00901	$\delta _{13}^{(2)}$	0.03833
$\delta _{14}^{(1)}$	0.00427	$\delta _{14}^{(2)}$	0.02056
$\nu ^{(1)}$	0.00114	$\nu ^{(2)}$	0.00085

### Numerical experiment

In this section, we propose a detailed description of the computational procedure for the proposed model. On 23 January 2020, a 56-year old man admitted to Toronto hospital emergency department in Toronto with a new onset of fever and nonproductive cough, and returning from Wuhan, China, the day prior^31,32^. It is believed this is the first confirmed case of 2019-nCoV in Canada, and according to the government report, the novel coronavirus arrived on the Canadian coast on January 25, 2020, first reported case. The above information suggests that the start date of the current pandemic in Canada is possibly xsto be January 22, 2020. Additionally, some research studies reported that the estimation of the incubation period of COVID-19 is from 2 to 14 days^1,33^. As a consequence, in the present study we consider 14 delays, $\tau _1 = 1$ day, $\tau _2 = 2$ days, $\ldots$, $\tau _{14} = 14$ days. Here we consider a calculation of 276 days, from January 22, 2020 to October 23, 2020. We decompose the time domain of 276 days into two parts : the time domain splitter is in the interval where the first wave is slowed down and the “second wave” begins, i.e. the splitter is in the interphase of two different scenarios. In this goal, we can choose the parameters ${\mathbf {p}}(t)$ as

9 $$\begin{aligned} \left\{ \begin{array}{lcl} {\mathbf {p}}(t) &{}=&{} {\mathbf {p}}^{(1)} \,\, \text {from January 22, 2020 to July 19, 2020}\, , \\ {\mathbf {p}}(t) &{}=&{} {\mathbf {p}}^{(2)} \,\, \text {from July 20, 2020 to October 23, 2020}\, , \end{array} \right. \end{aligned}$$

where ${\mathbf {p}}^{(1)} = (q, \alpha ^{(1)}, \beta ^{(1)}, \delta _1^{(1)}, \ldots , \delta _{14}^{(1)}, \nu ^{(1)})^T$ and ${\mathbf {p}}^{(2)} = (\alpha ^{(2)}, \beta ^{(2)}, \delta _1^{(2)}, \ldots , \delta _{14}^{(2)}, \nu ^{(2)})^T$ are some constants. The capability of an optimization package depends on the initial values of the parameters: for *q*, $\alpha$, $\beta$, $\nu$ we consider any positive random number less than unity, where as a choice of ${\varvec{\delta }}=(\delta _1, \ldots , \delta _{14})^T$ is tricky. For this purpose, we consider a vector of 14 positive random numbers ${\varvec{\delta }}$ such that $\delta _1< \cdots < \delta _4> \delta _5> \delta _6> \cdots > \delta _{14}$ and $\sum _{i = 1}^{14} \delta _i = 0.9$. We observe, from numerous numerical experiments, the renormalization factor 0.9 works perfectly for the computation.

For a complete calculation, we run the matlab code twice. Firstly, we run the code for the period January 22, 2020 to July 19, 2020 to obtain the estimated value ${\mathbf {p}}_{\text {est}}^{(1)}$ of ${\mathbf {p}}^{(1)}$, presented in Table 2, and the value of the error function $E({\mathbf {p}}_{\text {est}}^{(1)}) = 54.92$ for q = 0.005. Then, we run the code for the entire period from January 22, 2020 to October 23, 2020, but using the estimated value ${\mathbf {p}}_{\text {est}}^{(1)}$ for the interval January 22, 2020 to July 19, 2020, and obtain the estimated value ${\mathbf {p}}_{\text {est}}^{(2)}$ of ${\mathbf {p}}^{(2)}$ for the rest of the period, presented in Table 2, and the value of the error function $E({\mathbf {p}}_{\text {est}}^{(1)}, {\mathbf {p}}_{\text {est}}^{(2)}) = 48.38$, defined as

10 $$\begin{aligned} E( {\mathbf {p}}_{\text {est}}^{(1)}, {\mathbf {p}}_{\text {est}}^{(2)}) = \frac{1}{M}\sqrt{ \sum _{k = 1}^{M_1} (T^{(k)}( {\mathbf {p}}_{\text {est}}^{(1)}) - {\widetilde{T}}^{(k)})^2 + \sum _{k = M_1 + 1}^{M} (T^{(k)}( {\mathbf {p}}_{\text {est}}^{(2)}) - {\widetilde{T}}^{(k)})^2 } \,\,\, , \end{aligned}$$

where $M_1$ corresponds the date July 19, 2020.

## Conclusion

In this paper, we have derived a mathematical model based on a set of coupled delay differential equations, which was used to estimate the incubation period with good agreement with statistical works. Using the proposed model and publicly available data of confirmed cases, one could accurately estimate the incubation period in any region. We obtain the distribution of the incubation period from the population, so that it is better than any sample-dependent result. We have considered fourteen delays, but it is possible to consider an arbitrary number of delays. We can still use the model in the early stages of the disease, when the incubation period is unknown. We can select the maximum incubation period and then calculate the split confirmed corona cases (as shown in Fig. 2). The fit log-normal distribution of the split data with sufficiently small p-value will provide the good estimation. On the other hand, for example, suppose that the maximum incubation period is 16 days, and we consider 18 days. After calculation, we will only get few corona-positive cases for the incubation periods 17 and 18 days. After estimating the model parameters, one can estimate the incubation period of confirmed cases over a long period, over a small time interval, and even over a single day. The present approach can be used with a large-scale computation to estimate the recovery period of COVID-19, and to estimate the incubation period for different age-groups.
